# Dengue: Challenges for Policy Makers and Vaccine Developers

**DOI:** 10.1007/s11908-014-0404-2

**Published:** 2014-04-30

**Authors:** Annelies Wilder-Smith, Paul Macary

**Affiliations:** 1 Lee Kong Chian School of Medicine, Nanyang Technological University, Mandalay Road 11, Singapore, Singapore; 2 Department of Microbiology, Immunology Programme, National University of Singapore, Singapore, Singapore

**Keywords:** Dengue, Epidemiology, Economic evaluation, Chimeric dengue vaccine, Efficacy, Monotypic and heterotypic immunity, Immune correlates, Vaccine introduction, Vector control

## Abstract

Because of the increasing incidence, geographic expansion and economic burden of dengue transmission, dengue poses major challenges to policy makers. A vaccine against dengue is urgently needed, but vaccine development has been hampered by the lack of an appropriate animal model, poor understanding of correlates of successful human immunity, the fear of immune enhancement, and viral interference in tetravalent combinations. The most suitable target epitopes for vaccines, as well as the role of nonstructural proteins remain elusive. The chimeric yellow fever bone-based live attenuated dengue vaccine is furthest in development, but initial efficacy results have been disappointing. Lessons learnt from this failure will affect the design of future trials, and increase the urgency to identify the best epitope and immune correlates. Dengue vaccine introduction will not be the only strategy to combat dengue, but needs to be “packaged” with novel vector control approaches, with community-based interventions to reduce the number of breeding sites, and reducing the case fatality rate by improving case management.

## Introduction

The global dengue problem is worsening. Dengue, a mosquito-borne viral infection, is currently regarded as the most important arboviral disease internationally, as over 50 % of the world’s population live in areas where they are at risk of the disease [1]. Recent mapping of global dengue incidence suggests that an upper bound total of 3.97 billion people living in 128 countries are at risk of dengue globally, 824 million in urban areas and 763 million in periurban areas [2]. Estimates of the global incidence of dengue infections per year range between 50 million and 100 million. Accurate numbers are, however,– lacking due to poor disease surveillance, low levels of reporting, lack of inexpensive point of care diagnostic tests, and inconsistent comparative analyses [3•].

## Geographic and Numeric Expansion of Dengue

Using cartographic and consensus approaches that include inapparent and apparent infections, the estimate is closer to 400 million [2, 4••]. Even by more conservative estimates, dengue has increased 30-fold over the past 50 years [5]. Moreover, it has expanded into new areas. In Europe the first autochthonous cases were seen in southern France and Croatia in 2010, and the first major dengue epidemic occurred in Madeira, south of Portugal, in 2012 [6]. The extent of dengue in Africa remains unknown, but travelers have unmasked ongoing dengue outbreaks on several occasions [7–9] and dengue outbreaks have been reported in more than 22 countries in Africa in the past decade [7]. South East Asia and the Western Pacific region account for 75 % of the global dengue disease burden [10]. In the Americas, almost all countries now have hyperendemicity despite the absence of dengue in the middle of the 20th century [3•]. In the US, small outbreaks have occurred in Hawaii, Texas and Florida [11]. In international travelers returning from dengue endemic countries, dengue is now the second most common cause of fever [12–14]. A recent analysis by the GeoSentinel network, a global network of travel medicine providers, underpins the increasing trend of dengue seen in travelers [15•].

Why is dengue expanding so rapidly? The answer is important for policy makers in order to develop strategies to curb the problem. But the reasons are complex and involve almost all facets of modernization and globalization, and may include factors such as climate change where analyses of the impact are extremely challenging. Resurgence is most likely due to a multifactorial metric of unprecedented urbanization associated with overcrowding and open water sources, changes to the ecological habitat, lack of vector control, and virus evolution [16]. Emergence into new areas is mainly due to the increasing interconnectedness of our world through travel and trade [16, 17]. All these issues pose serious challenges to governments and policy makers.

## Increasing Socioeconomic Burden of Dengue

Dengue is characterized by high morbidity, but relatively low mortality, and this is one reason why it was neglected for so many decades. However, global deaths from dengue hemorrhagic fever already rank with those from yellow fever in exceeding combined deaths from all other viral hemorrhagic fevers, including Ebola, Marburg, Lassa, and Crimean-Congo [1]. Dengue represents a substantial economic and disease burden in South East Asia, a burden that is higher than that of 17 other conditions, including Japanese encephalitis, upper respiratory infections, and hepatitis B [18]. The estimated annual burden of dengue is 750,000 disability adjusted life years (DALYs) [18–21]. In most countries the main burden of this morbidity and mortality is carried by children [22–25]. Approximately 60 % of this cost is related to indirect or ‘productivity’ losses. In a study in Puerto Rico, hospitalized patients accounted for 63 % of the cost of dengue illness, and fatal cases represented an additional 17 % [26]. A study of 12 countries in South East Asia showed an annual economic burden of US $950 million, with approximately 52 % of these costs from productivity loss [18]. Dengue illness in the Americas was estimated to cost $2.1 billion per year on average (in 2010 US dollars), with a range of $1–4 billion in sensitivity analyses and substantial year to year variation [21].

## Challenges in Vector Control

The main arthropod vector for transmission of the dengue viruses (DENV) is *Aedes aegypti*, a mosquito species that is prevalent in both the tropics and subtropics [27]. The second, less effective vector, *Ae. albopictus*, is much more widely geographically distributed than *Ae. aegypti*, including in large parts of the US and southern Europe, but is the less efficient vector [28, 29]. Significantly, the *Aedes* mosquitos are predominantly active during daylight hours, which poses difficulties in controlling the vector–human interaction. The mosquito has a high affinity for human blood and high susceptibility to the four DENV serotypes. It is also well adapted to urban living and thrives in a peridomestic environment close to humans where it tends to persist because of its limited flight range [30]. *Aedes* takes a number of feeds per complete blood meal, often biting different people, which enhances virus transmission dynamics. Furthermore, *Ae. aegypti* distributes small numbers of eggs among many sites, and this “skip oviposition” is a driver of dispersal [31]. Skip oviposition also extends the duration of the gonotrophic cycle. Multiple oviposition sites limits the efficacy of attempts to suppress dengue transmission by source reduction and “focal” treatments with aerosols [31]. Innovative approaches that exploit or negate this behavior are required to ensure effective *Ae. aegypti* control. Lastly, another emerging problem is resistance against current standard vector control measures such as pyrethroids and temephos [32, 33]. Novel vector control strategies that involve releasing *Wolbachia*-infected mosquitos or genetically engineered mosquitoes appear promising [34, 35], but their use may be controversial and they are unlikely to be implemented at a large scale in the near future.

In summary, because of the increasing incidence and geographic expansion of dengue transmission, increasing socioeconomic burden and currently ineffective control strategies, a vaccine against dengue is urgently needed. But as discussed below, vaccine development poses even greater challenges, not only for scientists and vaccine developers, but also for policy makers and governments.

## Challenges in Dengue Vaccine Development

Dengue vaccines have been in development for over 50 years. Several vaccine technologies have been applied, including live attenuated virus, purified inactivated virus, recombinant subunits, virus-like particles and plasmid or viral vectors. Each of these approaches has its distinct advantages and disadvantages, and all are at different stages of development, as summarized in Table 1. Most of the live attenuated, inactivated and chimeric live vaccines have gone into clinical trials. The chimeric vaccine developed by Sanofi Pasteur (CYD) is the vaccine furthest in development.

**Table 1:** Approaches to the development of dengue vaccines, and their stages of development

Sponsor	Live	Inactivated	Viral replicon	DNA	Clinical phase		
					I	II	III
Sanofi Pasteur	Chimera yellow fever 17D–DENV (CYD)					✓	✓
Walter Reed Army Institute of Research (WRAIR)/Glaxo Smith-Kline (GSK)	Live attenuated virus (LAV) (PDK Passage)					✓	
Takeda	DENV–DENV chimera (DENV-2 PDK-53 backbone)				✓		
US National Institutes of Health (NIH)	Recombinant live attenuated; directed mutagenesis and DENV–DENV chimeras				✓		
Hawaii Biotech/Merck & Co.	Truncated recombinant E protein				✓		
Naval Medical Research Center (NMRC)				prM and E DENV-1, CMV promoter	✓		
WRAIR/GSK		Purified inactivated virus (PIV)			Planning		
GenPhar Inc.			Nonreplicating adenovirus-5 construct with prM and E DENV proteins				
Carolina Vaccine Institute			Venezuelan equine encephalitis virus replicon				

Dengue vaccine developers have to aim for a tetravalent vaccine that provides long-term broad protection against all four serotypes. But there are major scientific challenges which have slowed down their development. These include a lack of an appropriate animal model and a poor knowledge of correlates of successful human immunity to this virus versus correlates of immune pathology. Additional complications include a lack of understanding of the contribution of neutralizing antibodies to protection versus other immune mechanisms such as cytotoxic T cell responses, the fear of immune enhancement, a lack of understanding of the most suitable target epitopes for vaccines, an unknown role of nonstructural proteins such as NS1 in dengue immunity, viral interference of tetravalent combinations, and concern about reactogenicity in flavivirus-exposed individuals [1]. In the following, the challenges with regard to DENV per se and immunology are expanded further as these aspects are most important for understanding the lack of success so far with the dengue vaccine candidates with a particular focus on the chimeric dengue vaccine.

## Understanding the Complexity of Dengue Vaccine Development

DEN virions are composed of a lipid envelope modified by the insertion of envelope (E) proteins and premembrane/membrane (prM/M) proteins, with human antibodies mostly targeted at the E and prM proteins. However, the most suitable target epitopes for vaccine are still unknown. Anti-E antibodies may have higher type-specific neutralization capacity and lower ADE potential than anti-prM antibodies. The E protein of DENV is composed of three domains (EDI, EDII, EDIII) and is the main target of neutralizing antibodies. Epitopes in domain III of the E protein is involved in binding of DENV to cell receptors [36]. But most antibodies are specific for domain II of the E protein. Based principally on murine antidengue responses, ED III is currently still considered an antigenic target of importance for dengue vaccine development, but with more knowledge of E II, this notion may need to be modified and expanded [37]. Moreover, studies on human antibody repertoires for dengue have implicated complex conformational epitopes formed by E-protein homodimers and expressed on whole virions as targets for strongly neutralizing antibodies [38••, 39••].

Within each of the four DENV serotypes there are multiple genetic variations known as genotypes, reflecting the high degree of mutation and evolutionary pressure. Specific genotypes may have differences in viral fitness and be associated with more severe clinical phenotypes. It remains unclear whether identifying genotypic variation is important in dengue vaccine development, but a mismatch between the genotypic strains (Asian/American DENV-2 genotype in the vaccine strain) and the currently circulating divergent DENV-2 Asian 1 strains has been postulated to explain the low efficacy in the Thai phase 2b trial of the dengue chimeric vaccine [40••].

Natural protection against dengue infection is observed as monotypic, heterotypic and multitypic immunity [41••]. Solid and presumably life-long protection against reinfection with the same DENV ensues following a primary dengue infection, and this is called monotypic immunity [42]. Heterotypic immunity is a short-lived immunity against a new dengue serotype infection following a recent dengue infection. In other words, there is short-lived protection (up to 6 months, but mostly up to 3 months) in patients convalescing from a recent dengue infection if they are exposed to infection with another serotype. The shorter the interval, the greater the protection [43]. A similar phenomenon was observed on a large scale in Cuba where the severity of DENV-2 disease in DENV-2 immunes increased as the interval between infections increased from 4 to 20 years [44]. With shorter intervals, heterotypic immunity is protective, but with longer intervals it an increase in severity of disease occurs; a paradoxical challenge of the immunopathogenesis of dengue. Multitypic immunity is the notion that sequential dengue infections with more than two DENV in humans raise neutralizing antibodies to three or four DENV. Severe disease occurs during the second but not subsequent heterotypic DENV infections; in other words, disease severity is suppressed during a third or fourth DENV infection [41••].

The DENV-specific serum antibody response in humans consists of a large fraction of cross-reactive, poorly neutralizing antibodies and a small fraction of serotype-specific, potently inhibitory antibodies [38••]. Recent studies have shown that humans produce antibodies that neutralize DENV infection by binding a complex, quaternary structure epitope that is expressed only when E proteins are assembled on a virus particle [38••]. Mapping studies indicate that this epitope spans adjacent E protein dimers and includes the lateral ridge of domains I and II of the E protein. These results have significant implications for vaccine development.

The role of NS1 is also still unknown. Dengue NS1 interacts with the complement system and may directly contribute to the vascular permeability syndrome [41••]. There is an urgent need to study the role of dengue NS1 in the pathogenesis of severe disease and its role as a type-specific protective immunogen, as such an approach might safely bypass the risk of antibody-dependent enhancement [41••].

## Results from the First Efficacy Trial

Of the many dengue vaccine candidates, only the live-attenuated tetravalent, chimeric dengue–yellow fever vaccine developed by Sanofi Pasteur (CYD) has reached phase 2b and phase 3 clinical trials. This candidate is based on a yellow fever virus backbone with the yellow fever PrM and E genes replaced by DENV type-specific preM and E genes. The results of the phase IIb clinical trial from a single center in Thailand (CYD 23) were recently released [40••]. In this observer-masked, randomized, controlled, monocenter, phase 2b, proof-of-concept trial, 4,002 healthy Thai schoolchildren aged 4 – 11 years were randomly assigned (2:1) to receive three injections of dengue vaccine or control (rabies vaccine or placebo) at months 0, 6, and 12. The primary objective was to assess protective efficacy against virologically confirmed, symptomatic dengue occurring 1 month or longer after the third injection (per-protocol analysis). Virologically confirmed dengue occurred in 134 children during the study. Overall efficacy for all four serotypes was 30.2 % and differed by serotype with the efficacy for DENV-2 being only 9 %, despite the fact that neutralizing antibodies measured after doses 2 and 3 showed relatively high mean titers. Why did the CYD 23 trial show such poor clinical protective efficacy, particularly against DENV 2?

Halstead offers various hypotheses explaining the observed poor efficacy [41••]. Interference is a known problem in tetravalent live vaccine approaches. Interference is a failure of symmetrical production of neutralizing antibodies to each of the four DENV serotypes. Simultaneous administration of tetravalent CYD vaccine produces a hierarchy of immune response with DENV-4 showing the best response, and DENV-2 the lowest [42] which could explain the low protective efficacy against DENV-2. The lack of efficacy can also be explained by incomplete monotypic immune protection and/or heterotypic immune function. Heterotypic antibody may contribute to or prevent development of protective immune responses. Lastly, Halstead suggests incomplete multitypic immune protection. The absence of DENV T-cell immunity in CYD vaccines is the most likely reason for the lack of multitypic protective immunity, Halstead concludes [41••]. In addition, Sabchareon et al. hypothesized a mismatch between the vaccine strain and the genetically divergent circulating DENV-2 Asian 1 strain [40••].

## Lessons Learnt from the CYD 23 Trial

As Halstead writes, “the CYD 23 trial in Thailand is a cautionary tale for investigators designing future dengue vaccine efficacy trials” [45]. Several lessons can be learned from the failure of the CYD 23trial.

1. It may have been a wrong assumption that administering multiple doses of a tetravalent dengue vaccine that result in broadly cross-reactive DENV-neutralizing antibodies will provide protection comparable to that of sequential DENV infections. It is more likely that sequential monotypic DENV infections result in a different immune response than do sequential doses of a tetravalent vaccine [41••]. Perhaps a step by step immunization with one or more attenuated DENV or a prime-boost strategy would result in the type of protective immunity seen after sequential infections with two or more wild-type DENV [41••].
2. A good in vitro correlate for protection is still elusive, and this was the biggest set-back in the CYD 23 trial. The search for in vitro immune correlates of protection is now on. Halstead suggests to first characterize the immune responses in a monotypic challenge virus in a human controlled infection model, and then characterize the immune responses to monotypic vaccines [41••]. Neutralizing antibodies measured in Vero cells have failed as a good correlate of protection [46]. Halstead suggests that neutralizing antibodies could be studied using primary human monocytes instead [41••]. The use of a Fc-receptor-bearing cell system as an alternative to classical neutralization tests to studying vaccine-induced immunity has also been recommended by others [47].
3. Little or no attention was paid to the scale or form of the dengue-specific T-cell responses in the CYD 23 trial. The absence of DENV T-cell immunity in CYD 23 vaccines is the best current explanation for the lack of multitypic protective immunity seen in this trial [41••]. The role of nonstructural proteins in eliciting cellular immunity needs to be taken more seriously in other vaccine approaches. It is plausible that vaccines presenting yellow fever but not DENV nonstructural protein antigens such as the CYD vaccine do not mount a protective dengue T-cell response [41••]. Possibly a dengue–dengue chimeric vaccine that incorporates the nonstructural proteins of at least one of the four serotypes may be successful where the CYD vaccine failed. Formulations of chimeric dengue vaccine (DENVax) viruses containing the premembrane and envelope genes of serotypes 1–4 expressed in the context of the attenuated DENV-2 PDK-53 genome have shown good immunogenicity in animal and early human studies [48–50]. Currently, Takeda is developing this vaccine candidate.
4. The CYD 23 trial did not have sufficient numbers of subjects in whom the prevaccination immune status was known. It is important to include prevaccination immune status in future studies to evaluate the serological responses after vaccination [45]
5. If the hypothesis of Sabchareon et al. holds true about a mismatch between the vaccine strain and the circulating Thai strain [40••], then future dengue vaccine candidates will need to be composed of DENV strains closely matched to the circulating wild-type strains. If this is the case, a single dengue vaccine may not be suitable for global use and new vaccine virus strains will need to be introduced in response to viral evolution and genotypic variation [51].
6. Thomas and Endy suggest that a dengue human infection model (DHIM) should be added in the pathway of vaccine development [51]. This will not only help in identifying an immune correlate but also in down-selecting vaccine candidates, to make early vaccine formulation decisions, thus avoiding unnecessary costly large-scale trials.

## Conclusions: Challenges for Policy Makers and Vaccine Developers

A large number of diverse dengue vaccine candidates are in development ensuring continued innovation into the vaccine pipeline in addition to increasing competition leading to more affordable vaccines. The absence of an immune correlate of protection complicates dengue vaccine development efforts and regulatory strategies. The current vaccine pipeline does look promising, but major scientific hurdles still need to be overcome. Next generation dengue vaccines will need to take into account a favorable product profile including dose-scheduling, stability and efficacy [37]. Dengue human infection models will expedite vaccine development, despite their ethical challenges [51].

More accurate data are needed to inform the prioritization of research, health policy, and financial resources toward dengue control. The results of economic evaluations have often been conflicting because of the use of inconsistent assumptions. Health economic research specific to dengue is urgently needed to ensure informed decision making on the various options for controlling and preventing this disease [52]. Not only narrowly defined benefit categories such as healthcare cost savings, care-related productivity gains and health gains in reduction in morbidity and mortality through vaccination need to be taken into account, but also broader scopes such as outcome-related productivity gains, community health externalities, and community economic externalities [53•]. Specifically, the potential effects of dengue vaccination on outbreak control spending, income from tourism, foreign direct investment flows, and long-term economic productivity are important factors to consider in economic evaluations of dengue vaccination [53•].

The World Health Organization convened a consultation of experts to develop recommendations and guidelines for long-term safety assessment of live attenuated dengue vaccines [54••]. The Live Dengue Vaccines Technical Consultation Reporting Group has developed a risk-based approach in the long-term assessment of live dengue vaccines, particularly beyond the follow-up of subjects in pre-licensure clinical trials [54••]. They argue for a coordinated approach to developing a comprehensive and robust post-licensure safety database for dengue vaccines in parallel with their expanded use. Multiple stakeholders, including national public health, surveillance and regulatory authorities, national ethics committees, dengue vaccine developers, academia, clinicians and international donors will all need to be included in this coordinated approach. Their primary concern is also that severe disease due to vaccine failure versus vaccine-induced immune enhancement of disease are indistinguishable clinically and in currently available diagnostic assays. Hence benefit–risk assessments will have to rely on epidemiological studies during and after licensure [54••].

Evidence-informed introduction of vaccines into national programs, once a dengue vaccine is indeed licensed, poses another major set of challenges to policy makers. Governments need to decide upon the epidemiological threshold of dengue activity upon which a national dengue vaccination program is justified and cost effective. Should only high-risk groups or the total population be targeted, which age groups, when should catch-up programs be done? These are all challenging questions. Given that dengue mostly affects resource-limited countries, who will bear the massive expense related to vaccine introduction and to achieve and maintain high vaccination coverage? Changing the perception of dengue in non-endemic countries to help generate global support for dengue vaccination is paramount [55]. With the introduction of an effective vaccine, dengue is a disease that could be controlled, which means that in order to ensure a vaccine is introduced as rapidly as possible, there is a need to start preparing now [55].

Governments of countries that are currently unaffected by dengue need to be alert to the introduction of DENV via travelers and the emergence of autochthonous cases which can occur any time, in particular in countries where either *Ae. aegypti* or *Ae. albopictus* exist and where climate and ecological conditions are favorable [56]. The recent major dengue outbreak in Madeira, Portugal, only underlines this threat [57].

Mathematical models may enhance the understanding of population-wide dengue strategies, and can assess multiple intervention packages. Dengue vaccine introduction will in itself not be the only strategy to combat dengue. It needs to be a “public health package” that includes vector control, community-based interventions to reduce breeding sites, increasing the scope and breadth of surveillance, reducing the case fatality rate by improving case management, and perhaps novel entomological approaches to reducing transmission by altering mosquito ecology or genetics [58•]. The World Health Organization recommends further research into novel and community-based effective vector control interventions that correspond with the ‘Global Strategic Framework for Integrated Vector Management’ (IVM) of vector-borne diseases such as dengue [27, 59, 60]. An international multisectorial response, such as that outlined in the WHO Global Strategy for Dengue Prevention and Control, 2012–2020 [61], is now essential. To this end, the Partnership of Dengue Control (PDC) is a new organization led by Gubler that aims to utilize global expertise and resources for dengue control. Working closely with WHO on the Global Strategy for Dengue Control, the PDC’s goal is to accelerate innovation in vaccination, vector control, antiviral treatment, clinical management, diagnostics, surveillance, and social mobilization.
